# Assessment of adherence to therapy and exploring of barriers and facilitators in HIV positive patients in Tabriz-Iran: a mixed method study protocol

**DOI:** 10.1186/s12978-019-0766-x

**Published:** 2019-07-17

**Authors:** Amin Ghanbari, Maryam Ordibeheshti Khiaban, Armin Aslani, Amir Reza Faraji, Milad Mohammadi, Azita Fathnezhad Kazemi

**Affiliations:** 1 Student Research Committee, Islamic Azad University, Tabriz branch, Tabriz, Iran; 2 Department of Midwifery, Islamic Azad University, Tabriz Branch, Tabriz, Iran

**Keywords:** Adherence, Compliance, HIV, Mixed method

## Abstract

**Background:**

Adherence to therapy is a key predictor of the success of human immunodeficiency virus (HIV) treatment. There is limited information available from Iran about that and there is a need for more knowledge about factors influencing treatment adherence. The aim of this study is to examine adherence levels and to explore patients’ views about barriers and facilitators to HIV treatment adherence.

**Methods:**

This mixed-method study with the sequential explanatory design has two phases. The first phase (quantitative phase) is a cross- sectional study to assess the in Tabriz, the sixth large city of Iran. A convenience sampling method will be used to select 150 HIV positive patients who visit health centers in Tabriz. The second phase is a qualitative study designed to explore the HIV positive patients’ views of barriers and facilitators that can affect their adherence to therapy. In this phase, purposive sampling and in-depth individual interviews will be conducted for data collection. The conventional content analysis approach will be employed for data analysis. In addition to literature review and nominal group technique, the findings of the qualitative and quantitative phases, will be used to recommend some strategies to support adherence to therapy in HIV positive patients.

**Discussion:**

This is the first study looking into adherence to therapy and exploring of factors influencing in HIV positive patients which will be performed via a mixed-method approach, aiming to develop health practices improvement strategies. It is worth noting that there is no strategic guideline in Iran’s health system for improvement of treatment adherence in HIV positive patients. Health professionals and policy makers can be aware of factors influencing HIV treatment adherence. Therefore, it is hoped that the strategy proposed in the current study can lead to improvements their ability to treatment adherence.

## Plain English summary

The current study provides precise information about the adherence to therapy, and the factors influencing to it. This study is a mixed-method with the sequential explanatory design has two phases. The first phase (quantitative phase) is a cross-sectional study to assess the adherence to therapy and its relationship with age, sex, and Employment status who live in Tabriz- Iran. The second phase is a qualitative study designed explore patients’ views about barriers and facilitators to HIV treatment adherence. The findings of the qualitative and quantitative study in addition to literature review and nominal group technique will be used to recommend some strategies to support adherence to therapy to improve their health. The strategy proposed by this study may be helpful to treatment adherence and promote health them.

## Background

Acquired Immune Deficiency Syndrome (AIDS), as an emerging disease *in the* current century, is considered as the fourth leading cause of death worldwide [1, 2]. Over the past three decades, the pandemic caused by it has been threatened the whole world [3]. According to the World Health Organization (WHO), about 40.3 million people worldwide are infected with AIDS and new cases of infection in the world are rising [4]. Based on available statistics, so far, there are about 85,000 AIDS patients in Iran, and the incidence of this disease has increased in recent years, so that it has become one of the public health problems in the country [5, 6].

AIDS is of utmost importance as an important sociocultural phenomenon [7, 8]. Implementing therapeutic programs in an incomplete manner for various reasons can lead to faster disease progression in society and can increase care costs [2, 8, 9].

Due to increased access to antiretroviral drugs, including combination therapy in recent years, it is expected to prevent disease progression, improve quality of life and increase the lifespan of human life [10, 11]. Of course, the key to success is the willingness of HIV patients to adhere to combined antiretroviral regimens [12]. So adherence to treatment is a critical component of AIDS, and is considered as the most important determinant to improve disease and develop drug resistance [7, 13]. According to evidence, poor compliance with Antiretroviral Therapy (ART) is a major problem that leads to viral resistance, increase probability of admission and opportunistic infections and increase the risk of Human Immunodeficiency Virus (HIV) transmission [2, 14].

Sethi et al. during a study showed that those adhered to their prescribed treatment at 90–70% had a high drug resistance [15] . In fact, we are facing with all or none law in the adherence to treatment, and an adherence above 95% is required [15–17]. According to the results of Sethi et al., an increase of 10% in the adherence to treatment could lead to a 21% reduction in disease progression [15]. Based on the results of studies conducted in this field, various factors such as alcohol abuse and pessimism caused by the disclosure of disease are effective on the adherence to treatment [2, 9, 12]. Obstacles to the treatment adherence include lack of financial and food resources [8]. Cultural factors can be considered as factors involved in the lack of access and treatment to treatment [2, 12]. Investigating the status of the treatment adherence and obstacles and its facilitators will be essential for health planning and policy planning. There is a need for investigating the status quo due to increasing prevalence of HIV-positive people in the community and the lack of a study on the status of adherence to treatment and the effective factors on it.

## Objectives

The objectives of each phase are as follows:

### Objectives of the first phase: quantitative study

1. Determination of the adherence to therapy levels in HIV positive patient in Tabriz-Iran
2. Determination of the association between adherence to therapy with some Demographic characteristics (age, sex, Employment status and Income level) in HIV positive patient in Tabriz-Iran

### Objectives of the second phase: qualitative study

1. Exploration of the HIV patients’ views about barriers, facilitators in Tabriz-Iran
2. Provision of improvement strategies to adherence to therapy in HIV positive patient

## Methods/design

### Study design

**Fig. 1:**
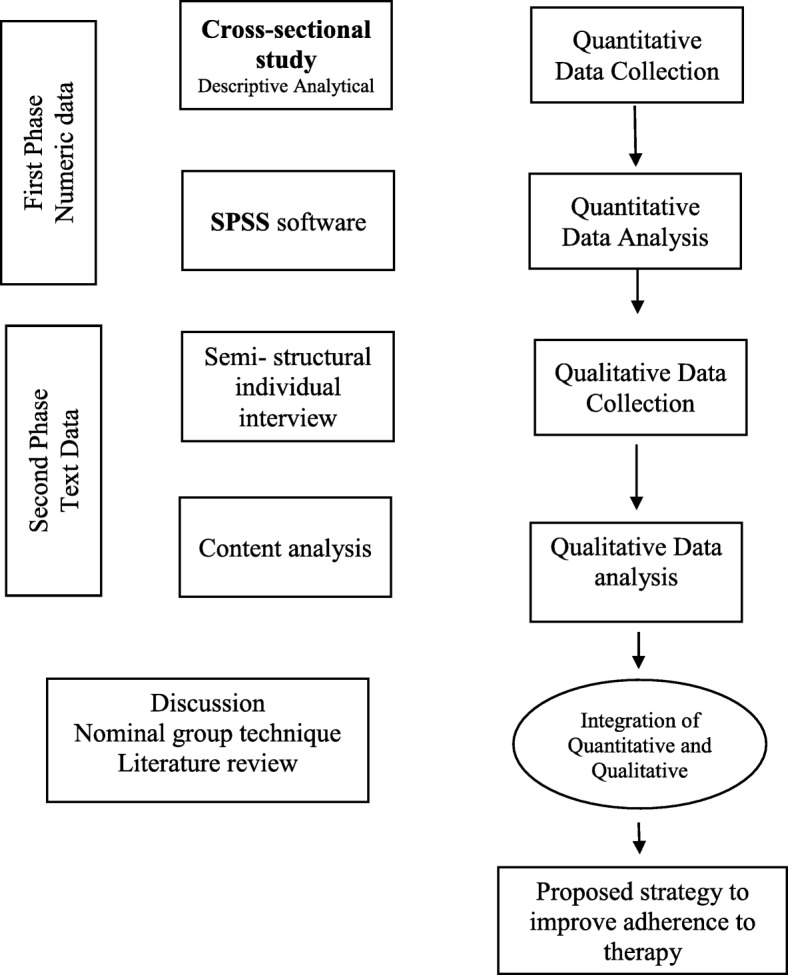
Study diagram

This study uses a mixed method with an explanatory sequential approach for data collection and analysis. The mixed-method paradigm is based on the principles and logic of pragmatism. According to this paradigm, a mixed use of qualitative and quantitative approaches results in a better understanding of the problem [18, 19]. The quantitative data will be collected in the first phase of the study. The second phase will include the collection and analysis of qualitative data. Then, qualitative and quantitative findings will be mixed in the stage of data interpretation and development of improvement strategies to adherence to therapy (Fig. 1).

### Phase I: quantitative study

First, a cross-sectional descriptive-analytical study will be conducted to evaluate adherence to therapy in HIV positive patient and the relevant factors.

### Sample size and sampling method

The sample size was calculated to be 138 based on the Sarna et al. study [14], Considering the 90% adherence to therapy, (d) of 0.05, α = 0.05 and power of 80% with Expressed by the formula. Regarding the Dropping samples 20%, the final sample size was determined to be 150. This study will be conducted in a health center which is the referral center for HIV patient in Tabriz affiliated with the Tabriz University of Medical Sciences.

$$ n=\frac{Z^21-\frac{a}{2}\cdot p\left(1-p\right)}{d^2} $$

Then, sampling will be made a convenience sampling method; and will continue to reach the calculated sample size. The researcher will explain the project to them via telephone (obtained from their medical records) and the eligible people will be invited to participate in the study.

### Inclusion criteria

Inclusion criteria for a patient to be included in the study were HIV-positive status, 18 year of age or older, ability to provide consent, living in Tabriz and having been on ART for at least 30 days.

### Exclusion criteria

Failure to complete the questionnaire completely.

### Scales and data collection

Quantitative data will be collected using the inclusion exclusion checklist, socio-demographic and, MMAS-8 questionnaire. The socio-demographic characteristics questionnaire will include age, sex, marital status, educational attainment, socioeconomic status and etc.

The Morisky Medication Adherence Scale MMAS-8 questionnaire, designed by Morisky and Wood 2008. The eight-item Morisky Medication Adherence Scale (MMAS-8) is a structured self-report measure of medication-taking behavior. The scale provides information on behaviors related to medication use that may be unintentional (e.g., forgetfulness) or intentional (e.g., not taking medications because of side effects) [20]. Besides its authors, other researchers have provided evidence of good psychometric properties of the scale [21, 22]. The MMAS-8 is currently available in 33 languages and is widely used in various types of studies [23]. This questionnaire consists of seven questions of two options (with yes or no), and a question as an answer. The overall scores range from zero to eight, with scores divided into three categories Poor drug compliance (score greater than 2), moderate adherence (score 1) And (2) and high adherence (zero score) [20]. The reliability and validity of the 8-item Morisky Medication Adherence Scale (MMAS-8) has been assessed with Moharamzad and etal in Iran. Internal consistency was acceptable with an overall Cronbach’s coefficient of 0.697 and test–retest reliability showed good reproducibility (*r* = 0.940); *P* < 0.001 [11].

### Data analysis

The quantitative data will be analyzed with SPSS-22. Sociodemographic and MMAS-8 questionnaire score will be described by frequency (percent), as well as mean (standard deviation) if the data are normally distributed or median (25 to 75 percentile) if they are not normally distributed. The relation between Medication Adherence and Sociodemographic characteristic will be determined using the independent test and Pearson correlation tests in the bivariate analysis. Then, the univariate logistic regression will be used to control confounding variables.

### Phase II: qualitative study

Second Phase is an exploratory qualitative study with a conventional content analysis approach to explore HIV positive patients’ views of barriers and facilitators about adherence therapy in more detail.

### Sampling method

The research participants will be selected through purposive sampling among HIV positive patient who have the tendency and ability to express and transfer concepts, with considering the maximum variability in terms of factors such as education, age, socioeconomic status. The withdrawal and non-attendance of the patient were considered as the exclusion criteria.

### Data collection

Qualitative data will be collected using in-depth and semi-structured interviews, containing open questions. Before conducting the qualitative phase, the desired items in the interview guideline will be designed based on the findings from the first phase and the relevant factors, and The interviewers piloted the interview on a subset of participants, and used this information to further refine the guide with respect to culturally sensitive and appropriate questions. The interview will begin with a key question, “current knowledge about medication functions and adherence”. Then, the interview will continue by presenting other questions, such as “frequency of missed medication doses and reasons for missing”; “probes regarding adherence facilitators”; “probes for specific suggestions of how adherence may be facilitated”.

The interview will continue with more in-depth items, such as “what do you mean? Why? Can you explain further? Can you give an example?” to explore the depth of their experience. During the interview, as far as possible, the Note field will be used and non-verbal data such as tone of voice and behaviors were recorded, too. The sampling will continue until data are saturated. All interviews will be carried out in care centers in a quiet room and without someone other than the interviewees.

### Data analysis

Data analysis process will be performed simultaneously with data collection using MAXQDA software version 10. The qualitative data will be analyzed using qualitative content analysis based on the Graneheim and Lundman method [24]. In this approach, the data will be analyzed through frequent text reading to obtain a full understanding of it. Then, the texts will be read word by word to extract the codes. First, the objective words that contain the key concepts will be specified. The researcher continued digging the text by taking notes from the initial analysis until the major codes will be extracted. In this process, the code labels reflecting more than one key thought will be directly extracted and specified. Then, the codes will be categorized based on their difference and/or relationships. The codes will be categorized into themes and main categories. Subcategories will be extracted based on differences and similarities.

### Validation

To validate the results, at first it will be tried to establish a friendly relationship with the participants. In order to increase the accuracy of the data and for verification of the accuracy of the data, after the registration, the interviews will be given to the participants to review and confirm their stated content and, if there will be any other content, it will be added to the data. Interviews will frequently read by the corresponding author of the paper; then, the text of the interviews with the extracted codes and categories will be shared with the colleagues and their comments will be used. External monitoring will be also used to increase the reliability. By providing the initial code derived from the analysis and examples of the extraction, as external observers, the concepts will be given to other researchers who will be not related to the study in order to determine whether they also will have a similar perception of the data or no.

### Integration of quantitative and qualitative data

To develop improvement strategies for improve adherence therapy in HIV positive patient, a comprehensive literature review will be carried out with a supportive approach to improve such practices. Following this, the results from qualitative and quantitative studies will be delivered to 10–12 experts. Then, their feedback and comments will be taken into account, using the nominal group technique.

## Discussion

Adherence to antiretroviral therapy is a key predictor of antiretroviral treatment success, and is potentially amenable to intervention [7, 12]. Sufficiently high levels of adherence to therapy are necessary to achieve and sustain viral suppression and to prevent disease progression and death, yet, many patients infected with human immunodeficiency virus (HIV) do not succeed in achieving or maintaining adequate levels of adherence to treatment [15, 22]. To this end, the status of this practice and its related factors should be identified. It is worth noting that there is no strategic guideline in Iran’s health system for improvement of treatment adherence in HIV positive patients. The current study provides precise information about the adherence therapy in HIV positive patient, and the its related factors and barrier and facilitator. This is the first study looking into adherence to therapy and exploring of factors influencing in HIV positive patients which will be performed via a mixed-method approach, aiming to develop health practices improvement strategies. Data collection through qualitative and quantitative methods contribute to better understanding of influencing factors at adherence therapy. The mixed-method approach focuses on Epistemological Pluralism. As a result, it supports the combination of opinions, approaches, and different, even contradictory, methods if they are helpful for understanding concepts. The strategy proposed by this study may be helpful in weariness of Health professionals and policy makers can be aware of factors influencing HIV treatment adherence so it can be improving medication adherence and helpful in promoting health in HIV positive patient. Therefore, it is hoped that the strategy proposed in the current study can lead to improvements their ability to treatment adherence.

## Data Availability

Not applicable.

## Abbreviations

AIDS: Acquired Immune Deficiency Syndrome
ART: Antiretroviral Therapy
HIV: Human deficiency Virus
MMAS: Morisky Medication Adherence Scale
WHO: World Health Organization
